# Gut microbiota has important roles in the obstructive sleep apnea-induced inflammation and consequent neurocognitive impairment

**DOI:** 10.3389/fmicb.2024.1457348

**Published:** 2024-12-06

**Authors:** Mingxing Tang, Yongliang Wu, Junyi Liang, Shuai Yang, Zuofeng Huang, Jing Hu, Qiong Yang, Fei Liu, Shuo Li

**Affiliations:** ^1^Department of Otolaryngology, Shenzhen Nanshan People’s Hospital, Shenzhen, China; ^2^Department of Otolaryngology, The 6th Affiliated Hospital, Shenzhen University Medical School, Shenzhen, China

**Keywords:** obstructive sleep apnea, intermittent hypoxia, systematic inflammation, neurocognitive dysfunction, gut microbiota

## Abstract

Obstructive sleep apnea (OSA) is a state of sleep disorder, characterized by repetitive episodes of apnea and chronic intermittent hypoxia. OSA has an extremely high prevalence worldwide and represents a serious challenge to public health, yet its severity is frequently underestimated. It is now well established that neurocognitive dysfunction, manifested as deficits in attention, memory, and executive functions, is a common complication observed in patients with OSA, whereas the specific pathogenesis remains poorly understood, despite the likelihood of involvement of inflammation. Here, we provide an overview of the current state of the art, demonstrating the intimacy of OSA with inflammation and cognitive impairment. Subsequently, we present the recent findings on the investigation of gut microbiota alteration in the OSA conditions, based on both patients-based clinical studies and animal models of OSA. We present an insightful discussion on the role of changes in the abundance of specific gut microbial members, including short-chain fatty acid (SCFA)-producers and/or microbes with pathogenic potential, in the pathogenesis of inflammation and further cognitive dysfunction. The transplantation of fecal microbiota from the mouse model of OSA can elicit inflammation and neurobehavioral disorders in naïve mice, thereby validating the causal relationship to inflammation and cognitive abnormality. This work calls for greater attention on OSA and the associated inflammation, which require timely and effective therapy to protect the brain from irreversible damage. This work also suggests that modification of the gut microbiota using prebiotics, probiotics or fecal microbiota transplantation may represent a potential adjuvant therapy for OSA.

## Introduction

Obstructive sleep apnea (OSA) is a chronic sleep-related breathing disorder that is characterized by recurrent collapses in the upper airway during sleep, directly causing sleep fragmentation (SF) and chronic intermittent hypoxemia (IH) (Lévy et al., 2015). The development of OSA is largely attributed to a narrow, high-arched hard palate, or midface hypoplasia with retro-positioning of the maxilla and chin, or an enlarged pharynx, in the majority of cases observed in individuals with obesity (Neelapu et al., 2017; Kubota et al., 2005). The structural disproportions would in turn bring the soft palate and tongue closer to the back of the throat, thus leading to partial or complete airway blockage. Apnea hypopnea index (AHI), which is defined as an average number of partial or full breathing stop events within an hour of sleep, is the most common used indicator for the OSA diagnosis and severity determination (Shahar, 2014; Pevernagie et al., 2020). A number of epidemiological studies based on AHI have revealed a high prevalence of OSA globally (Benjafield et al., 2019; Grote, 2019; Wei et al., 2022; Lv et al., 2023). Two consecutive works have demonstrated that the OSA incidence in the adult population of the USA is approximately 33% among males but lower among females (Benjafield et al., 2019). The overall prevalence of OSA among 38,000 Russian citizens is 48.9% (Khokhrina et al., 2020). Notably, studies covering China (Ding et al., 2022), Chile (Peñafiel et al., 2019), Canada (Dosman et al., 2022), Germany (Fietze et al., 2019), Switzerland (Heinzer et al., 2015), Singapore (Tan et al., 2016), and Japan (Nakayama-Ashida et al., 2008) revealed a higher incidence of OSA, all of them exceeding 50%. Therefore, these findings collectively indicate that OSA is the most prevalent disease diagnosed in the department of otorhinolaryngology. The typical symptoms of OSA often include snoring, breathing breaks, excessive daytime sleepiness, and dry mouth and headache upon waking (Veasey and Rosen, 2019; Gottlieb and Punjabi, 2020). More importantly, prolonged exposure of OSA patients to IH could activate systematic inflammation and impact central nervous system (CNS), which ultimately lead to brain structural injury and severe neurocognitive deficits. The precise mechanism by which inflammation is induced in the OSA condition remains poorly understood.

Herein, we provide an overview of the current state of the art, and discuss a hypothetical scenario in which OSA may directly alter the composition of the gut microbiota, elicit inflammatory responses, and consequently lead to neurocognitive impairment. This paper reviews progress from both clinical studies and animal models are included.

## Neurocognitive dysfunction is prevalent in OSA

Recent decades have borne witness to an increasing clarity regarding the prevalence of neurocognitive dysfunction among patients diagnosed with OSA. This phenomenon is characterized by deficits in attention, memory, and executive functions (Vanek et al., 2020; Xia et al., 2023; Kloepfer et al., 2009; Bawden et al., 2011; Hrubos-Strøm et al., 2012; Shieu et al., 2022). Indeed, clinical studies focusing on the effects of OSA have found that three distinct types of memory, including verbal, procedural and working memory, significantly decayed in the patients with OSA (Cunningham et al., 2023; Teh et al., 2023; Kloepfer et al., 2009; Naëgelé et al., 2006). Furthermore, several studies employed a more comprehensive set of tools to systematically identify cognitive impairment relevant to OSA (Xia et al., 2023; Bawden et al., 2011; Gnoni et al., 2023). Reviews of high quality are recommended to be consulted for a more detailed account of the cognitive impairment caused by OSA. This relatively underdiagnosed syndrome affects approximately 1 billion people globally and represents a significant public health concern (Benjafield et al., 2019).

In alignment with the abnormal cognitive function, structural alterations in brain tissues or regions have been identified through the utilization of diverse imaging technologies such as resting-state functional magnetic resonance imaging (fMRI) and computed tomography (CT). The affected areas across studies are diverse and often involved with multiple subregions (Zimmerman and Aloia, 2006), including the integrity of the gray or white matter (Lee et al., 2022; Castronovo et al., 2014), hippocampus (Kheirandish-Gozal et al., 2018; Macey et al., 2018; Gale and Hopkins, 2004), frontal lobe (Bai et al., 2021; Shu et al., 2022), temporal lobe (Shu et al., 2022; Morrell et al., 2010), cerebellum (Shu et al., 2022, Morrell et al., 2010), corpus callosum (Kheirandish-Gozal et al., 2018), and insular cortex (Kheirandish-Gozal et al., 2018). Despite the complexity and diversity, most of these areas are responsible for neurocognitive performance, indicating that structural alteration is a probable underlying cause of the observed deterioration in neurocognitive performance. For example, changes in the hippocampal volume have been identified in multiple studies through the MRI-based imaging analysis, while the hippocampus apoptosis or atrophy can cause learning, mnemonic, attentional, and executive function deficits. An early study, which involved with 17 newly diagnosed, untreated OSA patients and 15 age-matched healthy control subjects found that neurocognitive impairments were linked with a reduction of gray matter volume in the left hippocampus (entorhinal cortex), left posterior parietal cortex, and right superior frontal gyrus (Canessa et al., 2011). In another similar investigation, researchers identified atrophy of the neocortex and cerebellum, as well as a reduction in the volume of the hippocampal dentate gyrus and cerebellar dentate nucleus in patients with OSA (Kim H. et al., 2016). Of particular interest, after the continuous positive airway pressure (CPAP) treatment was administered to the patient cohorts in both studies, the impaired brain structure was restored to normality together with improved cognitive function, indicative of the reversibility of cognition deficits.

The intimate association of OSA with neurocognitive dysfunction has been also observed in a range of animal models, including pigs (Lonergan et al., 1998), dogs (Hendricks et al., 1993; Hendricks et al., 1987), rabbits (Schiefer et al., 2020; Yu et al., 2014), cats (Neuzeret et al., 2011), rats (Nácher et al., 2007; Farré et al., 2003), and mice (Qiu et al., 2023; Puech et al., 2022; Veasey et al., 2013; Nair et al., 2011a; Zhu et al., 2007; Puech et al., 2023). The disease has been modeled using either sleep fragmentation (SF), or intermittent hypoxia (IH), or both. SF can be induced by sleep disruption with experimental devices (Nair et al., 2011b; Ramesh et al., 2012), while IH is triggered by repeated exposure to lower oxygen levels (Badran et al., 2020; Puech et al., 2022). Although adverse effects of IH and SF on cognitive function may differ, common outcomes include impaired sleep quality, abnormal behavior, reduced learning ability and impaired physical functioning. In a study based on a rat model of OSA, for example, exposure to IH resulted in deficits in spatial memory and learning performance as assessed by Morris water maze tasks, together with the hippocampal apoptosis (Gao et al., 2017).

## Inflammation caused by OSA is very likely to induce cognitive deficits

The pathogenesis of cognitive impairment induced by OSA is believed to be complex and remains poorly elucidated (Lv et al., 2023; Liu et al., 2020; Orrù et al., 2020). However, several lines of evidence from both clinical and animal model studies strongly support involvement of inflammation. Firstly, numerous studies have found that OSA can cause a systematic or local inflammation, as evidenced by increased levels of serum inflammatory cytokines are often observed in OSA patients (Liu et al., 2020; Nadeem et al., 2013; Bouloukaki et al., 2017; Bozic et al., 2018; Motamedi et al., 2018; Svatikova et al., 2003; Sozer et al., 2018). Tumor necrosis factor (TNF)-*α* and interleukin (IL)-6 are two representative biomarkers that are closely linked with OSA (Kheirandish-Gozal and Gozal, 2019), and more intriguingly both are also thought to contribute to neurocognitive dysfunction (Tegeler et al., 2016). Secondly, it is frequently observed that the activation of inflammatory processes and cognitive deficits occur concurrently in a considerable number of rodent models of OSA induced by IH (Liu et al., 2020; Dong et al., 2018; Sapin et al., 2015; Shi et al., 2018; Snyder et al., 2017; Darnall et al., 2017; Kim et al., 2013; Smith S. M. et al., 2013; Deng et al., 2015). Thirdly, in clinical studies, the magnitude of inflammation, as indicated by serum levels of various inflammatory cytokines, is frequently correlated with the severity of OSA (Nadeem et al., 2013; Sozer et al., 2018; Bouloukaki et al., 2017; Bozic et al., 2018; Motamedi et al., 2018). For instance, a recent analysis involving 858 OSA patients and 190 matched controls demonstrated that the serum levels of uric acid and high-sensitivity C-reactive protein (hsCRP), two markers of inflammation, were elevated in the severe group compared to the mild group (Bouloukaki et al., 2017). Fourthly, effective OSA therapy by CPAP can improve neurocognitive performance and also reduce/resolve inflammation (Tichanon et al., 2016; Lu et al., 2017; Ohga et al., 2003; Kuramoto et al., 2009; Wu et al., 2010; Jin et al., 2017; Yokoe et al., 2003; Steiropoulos et al., 2009; Schiza et al., 2010). Altogether, ample evidence from these works substantiates the pivotal role of OSA-induced neuroinflammation in the pathogenesis of neuronal injury and subsequent cognitive deficits.

The precise mechanistic pathway by which OSA triggers inflammation remains poorly understood. However, it has been postulated that HIF-1α, a critical transcription factor responsive to hypoxic conditions, is activated to increase ROS synthesis, which would in turn initiate oxidative stress and the inflammatory process (McGettrick and O’Neill, 2020). Furthermore, there is also evidence to show that chronic IH conditions observed in OSA patients stimulate leptin, an obesity biomarker in white adipose tissue (Pan and Kastin, 2014), while leptin can further promote production of proinflammatory cytokines (Berger and Polotsky, 2018). These changes may further lead to monocyte-endothelial cell adhesion, dysfunction of endothelial cells, breach of the blood–brain barrier, and finally the perfusion of inflammatory cytokines and macrophages into the central nervous system. Consequently, the excessive neuroinflammatory response results in the activation of glial cells, synaptic damage and loss, neuronal necrosis and apoptosis, and ultimately a significant exacerbation of neurocognitive deficits (Liu et al., 2020).

## OSA altered gut microbiota

In addition to the aforementioned effects on inflammation and cognitive function, the impact of OSA can even extend to the gastrointestinal tract to modulate the oxygen concentrations and further the ecosystem, where at least 100 trillion bacteria colonize (Honda and Littman, 2016; Rooks and Garrett, 2016; Gomaa, 2020). In light of the existence of an oxygen concentration gradient in the range of 150–200 μm near the gut epithelium (Espey, 2013) and the susceptible responsiveness of gut microbiota to oxygen level change (Albenberg et al., 2014), it seems highly probable that chronic exposure to hypoxia would favor the survival of obligate anaerobes and therefore alter the bacterial diversity. Indeed, several mouse model-based studies have demonstrated that IH intervention can induce a periodic hypoxia pattern in the arterial blood and the lumen of the small intestine, as well as an increased abundance of obligate and facultative anaerobes (Moreno-Indias et al., 2015; Khalyfa et al., 2021; Lucking et al., 2018; Durgan et al., 2016), despite the possibility that IH-induced systemic immune responses may exert a modulatory effect on gut microbiota. Another important feature of OSA is nocturnal arousal due to sleep fragmentation. Interestingly exposure of mice to sleep fragmentation also caused notable change in gut microbiota, characterized by an increase in *Firmicutes* and a decrease in *Bacteroidetes* at the phylum level (Poroyko et al., 2016). It is noteworthy that a gut dysbiosis was also a prevalent trait in OSA patients. Two clinical studies investigating the OSA patients from disparate regions in China identified an altered gut microbiota profile (Ko et al., 2019; Wang et al., 2022). Furthermore, the composition of the gut microbiota was found to be significantly altered in pediatric patients with OSA in comparison to their age-matched healthy controls (Valentini et al., 2020; Collado et al., 2019). Consistently, an interesting single-armed study that investigated the responses of nine normal-weight men under two occasions, either with two nights of normal sleep or two nights of partial sleep deprivation. It revealed that sleep loss can directly induce an increased *Firmicutes* to *Bacteroides* (F/B) ratio in gut microbiota (Benedict et al., 2016).

## Gut microbiota composition alteration associated with inflammatory activation in OSA patients

In light of the pivotal roles of gut microbiota in regulating human physiology, particularly immunity (Kamada et al., 2012; Donohoe et al., 2011; Zheng et al., 2020; Belkaid and Harrison, 2017; Albhaisi et al., 2020; Morais et al., 2021), a hypothesis was therefore proposed that the OSA-induced gut dysbiosis, often featured with a changed F/B ratio, might contribute to inflammation, and potentially the cognitive dysfunction. In the context of the microbiota-immune system interaction, multiple microbial metabolites and components, including short-chain fatty acids (SCFAs), lipopolysaccharide (LPS) and exotoxins, act as potent effectors, facilitating a bridge between gut microbiota and local or systematic immunity (Rooks and Garrett, 2016; Wang G. et al., 2019). Indeed, the immunological effects of these microbiota-derived molecules are manifold, encompassing both innate and adaptive immunity (Tang et al., 2021). SCFAs are primarily produced from indigestible oligosaccharides by some beneficial members of the *Bacteroidetes* phylum, including the families *Lactobacillaceae*, *Ruminococcaceae*, *Erysipelotrichaceae*, *Bifidobacteriaceae*, and *Clostridium* (den Besten et al., 2013). In addition to serving as a source of energy for intestinal epithelial cells (IEC) (Rivière et al., 2016), SCFAs have pleotropic roles in the fortification of the gut barrier and maintenance of immune homeostasis. More specifically, these beneficial regulatory actions include stimulation of mucus production (Wrzosek et al., 2013), regulation of tight junction (TJ) proteins via multifaceted signaling pathways (Parada Venegas et al., 2019), polarization of anti-inflammatory macrophages (Ji et al., 2016), increased production of antimicrobial peptide (AMP) (Qiu et al., 2012), activation of NLR-family-pyrin-domain-containing-3 (NLRP3) inflammasomes and production of homeostatic cytokine interleukin-18 (IL-18) (Macia et al., 2015), modulation of B cell differentiation and Immunoglobulin A (IgA) secretion (Kim M. et al., 2016), reduced expression of T cell-activating molecules on antigen-presenting cells (Park et al., 2015), and increased number and function of colonic regulatory T (T_regs_) cells (Smith P. M. et al., 2013). In contrast, several gut microbial members, including *Desulfovibrio, Prevotella, Lachnospiraceae,* and *Paraprevotella*, have been demonstrated to induce inflammatory responses and disrupt the structural integrity of the gut barrier. LPS is a well-known bacterial endotoxin with profound immunostimulatory and inflammatory capacity. Contrary to the beneficial effects of SCFAs, LPS has the potential to bind to its cognate toll-like-receptor (TLR4) and promote the activation of inflammatory macrophage (M1 polarization), which in turn leads to the production of an array of inflammatory cytokines, IL-1β, IL-6, IL-12, and TNF-*α* (Martinez et al., 2008). Through the same signal pathway, LPS can also compromise the integrity of the intestinal barrier (Guo et al., 2013). Furthermore, the prevalence of Prevotella and Desulfovibrio, which possess mucin-degrading capabilities, was found to be elevated in individuals with OSA, thereby exacerbating the gut permeability. An increase in those bacterial species will therefore cause the leakage of LPS and other bacterial components from the gut into the blood circulation, thus stimulating the release of inflammatory mediators and aggravating systemic inflammation.

Consistent with the above analysis, an investigation into the alteration of the gut microbiota in OSA patients indeed validated the abundance reduction in the abundance of bacteria associated with SCFA production, or alternatively an increase in the abundance of pathogenic ones, despite the fact that only a limited number of clinical studies in this direction existed. One study involving 93 OSA patients revealed that alterations in the gut microbiota were characterized by a decrease in the abundance of SCFA producers (including *Faecalibacterium, Bifidobacterium, Lactobacillus*, and *Bacteroides*), an increase for the pathogenic *Prevotella*, and concomitantly a reduction in the serum levels of IL-6 (Ko et al., 2019). Interestingly, this work did not identify a profound change in the F/B ratio between the OSA patients and healthy controls (Ko et al., 2019), suggesting that the F/B ratio alteration may not be a general OSA-associated rule, and that a more detailed analysis of specific bacterial taxa level should be necessary. In another observational study, the inflammation-related bacteria (*Megamonas, Lactobacillus, Megasphaera*, and *Coprococcus* at the genus level) were also enriched in OSA patients, while *Alistipes*, *Eubacterium coprostanoligenes, Blautia, Roseburia, Fusobacteria,* and *Ruminococcus gnavus* were found to be depleted. The pro-inflammatory cytokine IL-1β and TNF-α were elevated with OSA (Lu et al., 2022). A comparable pattern was identified among the pediatric OSA patients, whereby the relative abundances of several well-documented SCFA producers, including *Bacteroides, Bifidobacterium, Ruminococcus, Collinsella,* and *Faecalibacterium*, exhibited a decline (Valentini et al., 2020). The hypothesis was further validated in the two clinical trials, in which healthy human subjects were exposed to either a short-term or a long-term period of sleep deprivation, directly resulted in a reduction of the abovementioned species with SCFA-production capacity (Gao et al., 2023; Benedict et al., 2016).

Moreover, a comparable pattern was observed in multiple OSA animal model-based gut microfloral analyses, with an increase in pathogenic microbes being a particularly prevalent finding. For example, *Prevotella, Paraprevotella, Desulfovibrio*, and *Lachnospiraceae* were found to be enriched in a mouse model of OSA, induced by either SF or IH (Gao et al., 2023; Poroyko et al., 2016; Badran et al., 2020; Khalyfa et al., 2021; Badran et al., 2023; Yan et al., 2024). Interestingly, treatment of the sleep-deprived mice with butyrate, a type of SCFA, significantly ameliorated intestinal mucosa injury and inflammation response through the suppression of the HDAC3–GSK-3β–Nrf2–NF-κB signaling cascade (Gao et al., 2023). In another study, neonatal brain immaturity, white matter injury (WMI), reduced abundance of beneficial gut microbes *Bacteroides thetaiotaomicron* and *Parabacteroides distasonis* and accumulation of microbiota-derived cholic acid were identified in chronically hypoxic rats, whereas administration of *B. thetaiotaomicron* and *P. distasonis* reverted the cholic acid concentration and rescued the chronic hypoxia-induced WMI and inflammation. These findings suggest that the administration of SCFAs or probiotics may represent a potential strategy for alleviating the inflammation and neurocognitive deficits associated with OSA (Yan et al., 2024).

Fecal microbiota transplant (FMT) simply implies the transfer of stool samples from a donor’s colon to a recipient’s colon (Gupta and Khanna, 2017). FMT has demonstrated considerable promise in the treatment of intestinal infection, inflammatory bowel disease, hypertension, obesity, and diabetes mellitus (Kelly et al., 2015; Turnbaugh et al., 2006; Adnan et al., 2017; Wang H. et al., 2019). Although only a few studies have employed FMT in analyzing the basis of OSA, two recent works showed that FMT from the IH-mice can elicit inflammation and neurobehavioral disorders in naïve mice (Badran et al., 2020; Poroyko et al., 2016). This provides further robust evidence that gut microbiota dysbiosis is, at least partially, a causal basis of cognitive dysfunction.

## Conclusions and perspectives

**Figure 1:**
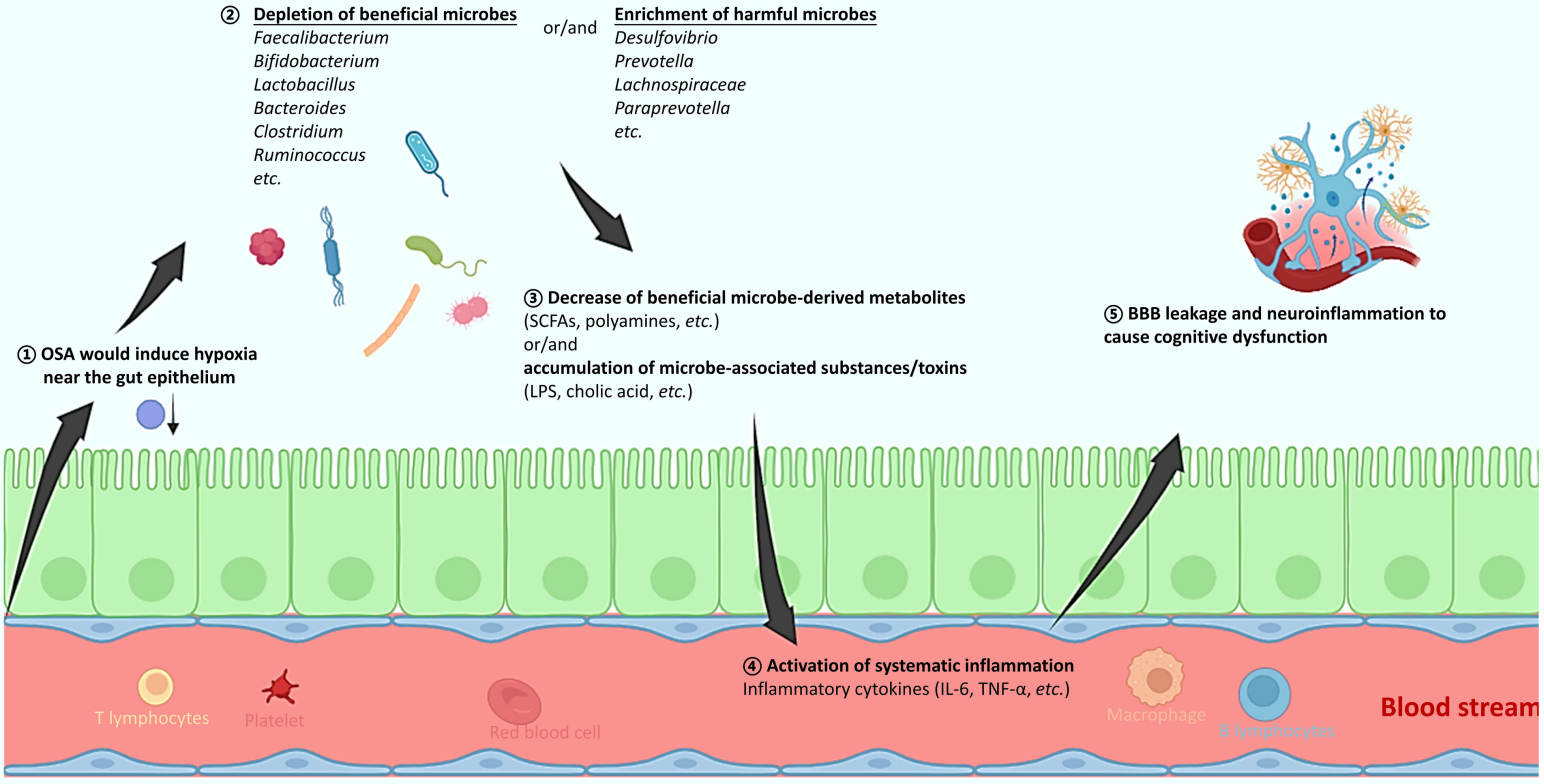
A schematic diagram explaining how obstructive sleep apnea (OSA) would alter the gut microbiota, activate systematic inflammation, and consequently induce brain tissue injury and cognitive dysfunction. (1) The OSA-induced hypoxia might alter the oxygen concentration near the gut epithelium. (2) The depleted oxygen radiation thus favors the increased abundance of obligate and facultative anaerobes, resulting in the gut microbiota change that is mostly featured with an increased ratio of *Firmicutes* to *Bacteroides* (F/B). Specifically, producers of beneficial metabolites such as SCFAs and polyamines decrease, while microbial members with pathogenic potentials increase. (3) The gut microbiota composition change further leads to accumulation of microbial toxins, and/or decrease of a wide spectrum of beneficial metabolites. (4) The gut barrier function might be compromised, and levels of various inflammatory cytokines are elevated, resulting in a systematic inflammation. (5) The inflammatory cytokines can breach the BBB, activate microglial cells, cause neuroinflammation, and consequently result in cognitive deficits. OSA, obstructive sleep apnea; SCFAs, short chain fatty acids; LPS, lipopolysaccharide; IL-6, interleukin-6; TNF-α, tumor necrosis factor-α; BBB, blood brain barrier.

The prevalence of OSA is markedly high across countries and regions, and it is frequently complexed with a variety of comorbidities. With firm evidence from both clinical studies and animal models, the present review reveals a clear and compelling link of OSA with alterations in the gut microbiota, systematic inflammation, brain substructural changes and neurocognitive impairment, respectively. A hypothesis is therefore proposed to explain the pathogenesis of OSA that chronic IH or SF in OSA patients would trigger gut microbiota dysbiosis, which is often characterized by depletion of producers of beneficial microbial metabolites, or/and enrichment of microbes with potentials to impair mucosa and activate inflammation (Figure 1). This in turn results in the activation of systematic inflammation, with inflammatory cytokines breaching the blood–brain barrier, activating microglial cells, and ultimately causing necrosis and apoptosis of neural cells and neurocognitive deficits. It is imperative to emphasize the importance of timely diagnosis and treatment of OSA and its associated inflammation, in order to prevent or alleviate the irreversible neurocognitive damage. Moreover, the modulation of gut microbiota using prebiotics (such as butyrate), or probiotics (SCFA producers) may represent a potential and effective adjuvant therapy for OSA. More future works in this direction are still needed.
